# MiR-21 and MiR-155 promote non-small cell lung cancer progression by downregulating *SOCS1*, *SOCS6*, and *PTEN*

**DOI:** 10.18632/oncotarget.13022

**Published:** 2016-11-02

**Authors:** Xinying Xue, Yuxia Liu, Yong Wang, Mingming Meng, Kaifei Wang, Xuefeng Zang, Sheng Zhao, Xiaohua Sun, Lei Cui, Lei Pan, Sanhong Liu

**Affiliations:** ^1^ Department of Special Medical Treatment-Respiratory Disease, Beijing Shijitan Hospital, Capital Medical University, Beijing, China; ^2^ Department of Respiratory Diseases of Chinese PLA General Hospital, Beijing, China; ^3^ Department of Research, Peking Union Medical Collage Hospital, Beijing, China; ^4^ Department of Gastroenterology, Beijing Shijitan Hospital, Capital Medical University, Beijing, China; ^5^ Department of Intensive Care Unit, Beijing Shijitan Hospital, Capital Medical University, Beijing, China; ^6^ Department of Cardiology, Peking University Ninth School of Clinical Medicine, Beijing Shijitan Hospital, Beijing, China; ^7^ Institute of Health Sciences, Shanghai Institutes for Biological Sciences, Chinese Academy of Sciences and Shanghai Jiao Tong University School of Medicine, Shanghai, China; ^8^ Department of Central Laboratory, Beijing Shijitan Hospital, Capital Medical University, Beijing, China; ^9^ Shanghai Institute of Advanced Immunochemical Studies, ShanghaiTech University, Shanghai, China

**Keywords:** non-small cell lung carcinoma, miR-21, miR-155, SOCS1, SOCS6

## Abstract

Lung cancer remains the leading cause of cancer-associated death worldwide. MiR-21 and miR-155 are the most amplified miRNAs in non-small cell lung carcinoma (NSCLC), and are critical promoters of NSCLC progression. However, it remains unclear how miR-21 and miR-155 induce cancer progression, and whether these miRNAs share common targets, such as tumor suppressor genes required to prevent NSCLC. Here we report that miR-21 and miR-155 levels are elevated in NSCLC and are proportional to the progression of the disease. In addition, miR-21 and miR-155 share nearly 30% of their predicted target genes, including *SOCS1*, *SOCS6*, and *PTEN*, three tumor suppressor genes often silenced in NSCLC. Consequently, antagonizing miR-21, miR-155 or both potently inhibited tumor progression in xenografted animal models of NSCLC. Treatment with miR-21 and miR-155 inhibitors in combination was always more effective against NSCLC than treatment with a single inhibitor. Furthermore, levels of miR-21 and miR-155 expression correlated inversely with overall and disease-free survival of NSCLC patients. Our findings reveal that miR-21 and miR-155 promote the development of NSCLC, in part by downregulating *SOCS1*, *SOCS6*, and *PTEN*. Combined inhibition of miR-21 and miR-155 could improve the treatment of NSCLC.

## INTRODUCTION

Lung cancer is the most common cause of cancer-related death worldwide [1]. Non-small cell lung cancer (NSCLC), which encompasses adenocarcinoma, squamous cell carcinoma and large cell carcinoma, accounts for over 80% of all lung cancer cases [2]. Despite years of research, the prognosis for patients with lung cancer remains dismal, and less than 15% of diagnosed patients survive longer than 5 years [3].

Suppressor of cytokine signaling 1 (SOCS1) can significantly induce apoptosis and suppress the growth of lung cancer cells [4]. Suppressor of cytokine signaling 6 (SOCS6) is widely expressed in numerous tissues and is down-regulated in many cancers, including lung, colorectal, gastric, ovarian, stomach, thyroid, hepatocellular, and pancreatic cancer [5]. Reduced copy numbers and mRNA levels of *SOCS6* are associated with disease recurrence in primary lung cancer, and thus may serve as useful prognostic biomarkers [6]. In addition, some studies have demonstrated that phosphatase and tensin homolog (PTEN) functions as a tumor suppressor in NSCLC [7, 8]. Although these proteins have been implicated in NSCLC, it is not understood how the genes encoding them are regulated during NSCLC progression.

MicroRNAs (miRNAs) are a class of small non-coding RNAs that post-transcriptionally repress target genes by binding their 3′-untranslated regions (3′-UTRs) [9]. The most frequently amplified miRNAs in lung cancer are miR-21, miR-155, miR-30d, and miR-17 [10], so these miRNAs also may be promising prognostic markers of lung cancer. In particular, the levels of miR-21 and miR-155 can be used to predict recurrence and poor survival in NSCLC [11]. Moreover, the gene expression of the tumor suppressor *PTEN* is downregulated by several miRNAs in NSCLC, including miR-21, miR-214, miR-205, miR-92, miR-106, and miR-10a [7, 12–16].

While several studies have focused on the correlation of miR-21 or miR-155 with the survival of patients with NSCLC, there has been no report investigating how miR-21 and miR-155 together contribute to the progression of this disease. One important issue that needs to be emphasized is that drug treatments inhibiting miR-21 or miR-155 alone have not significantly inhibited NSCLC progression. We reason that this may be because certain tumor suppressors are targets of both miR-21 and miR-155; thus, when miR-21 alone is inhibited pharmaceutically, the expression of its target genes might not be restored because these target genes are still being suppressed by miR-155, which is highly expressed in cancer cells. Therefore, it is necessary to inhibit both miR-21 and miR-155 in order to restore the function of tumor suppressor genes.

Here, we investigate how miR-21 and miR-155 regulate NSCLC progression by evaluating their binding to predicted target genes, and by testing single and combined inhibitors of these miRNAs for their effects on tumor growth.

## RESULTS

### Upregulation of miR-21, miR-155, miR-214 and downregulation of SOCS1, SOCS6, and PTEN in NSCLC

According to previous data, miR-21, miR-155 and miR-214 are the most amplified miRNAs in human lung cancer. To investigate whether these miRNAs synergistically induce lung cancer progression by binding to common targets, we compared their predicted targets using the TargetScan database (www.targetscan.org). In total, 419 overlapping targets were identified (Supplementary Figure S1). Notably, the regulation pathways of the predicted targets of miR-21, miR-155 and miR-214 were mostly related to human cancers, including NSCLC (Supplementary Figure S2) (http://cpdb.molgen.mpg.de/). After using other methods of target prediction (www.microrna.org), and taking into consideration the previously reported downregulation of SOCS1, SOCS6 and PTEN in NSCLC, we selected these three genes for further investigation in this study.

**Figure 1:**
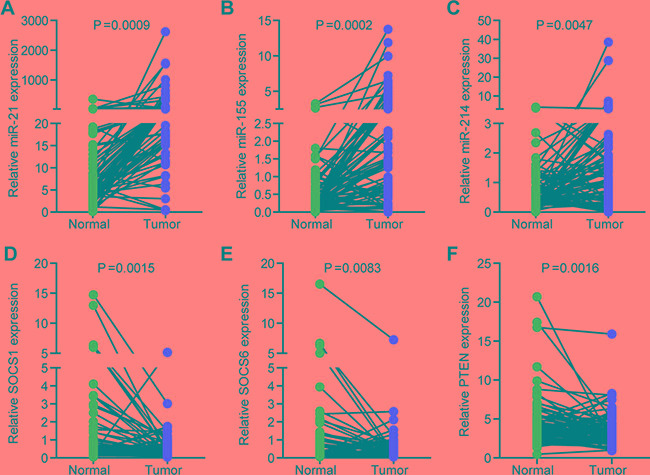
The expression of miR-21, miR-155, miR-214, and their targets, SOCS1, SOCS6, and PTEN, in NSCLC (**A**–**C**) The expression of miR-21 (A), miR-155 (B), and miR-214 (C) in 80 paired human normal and NSCLC samples. U6 was used as an internal normalization control. (**D**– **F**) The expression of SOCS1 (D), SOCS6 (E), and PTEN (F) in 80 paired human normal and NSCLC samples. GAPDH was used as an internal normalization control.

We then examined the expression of miR-21, miR-155 and miR-214 in 80 paired human normal and NSCLC samples. The levels of miR-21, 155 and miR-214 were significantly greater in NSCLC samples than in paired normal tissues (Figure 1A–1C). Concomitantly, *SOCS1*, *SOCS6*, and *PTEN* were downregulated in NSCLC samples (Figure 1D–1E).

### The levels of miR-21, miR-155, miR-214, and their target genes, *SOCS1, SOCS6*, and *PTEN*, are associated with NSCLC disease stages

**Figure 2:**
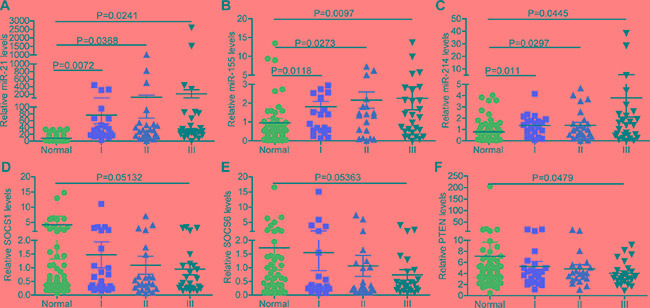
The expression of miR-21, miR-155, miR-214, and their targets, *SOCS1*, *SOCS6*, and *PTEN*, in different stages of NSCLC (**A**–**C**) The expression of miR-21 (A), miR-155 (B), and miR-214 (C) in different stages of NSCLC. (**D**–**F**) The expression of *SOCS1* (D), *SOCS6* (E), and *PTEN* (F) in different stages of NSCLC. (Sample size: normal = 80, stage I = 24, stage II = 24, stage III = 32).

To evaluate the expression of miR-21, miR-155 and miR-214 in different stages of NSCLC, we divided 80 paired human normal and NSCLC samples into three groups according to the disease stage. There were 24, 24 and 32 samples in stages I, II and III, respectively. The expression of miR-21, miR-155 and miR-214 gradually increased as NSCLC progressed (Figure 2A–2C), whereas the gene expression of *SOCS1*, *SOCS6*, and *PTEN* gradually decreased (Figure 2D–2F). Furthermore, in a target prediction analysis, the regulatory networks of miR-21, miR-155, miR-214, miR-21/155/214 included overlapping targets with different patterns (Supplementary Figure S3). For example, miR-155 had a more complicated regulatory network than miR-21 or miR-214 (Supplementary Figure S3B). In addition, miR-21 was more profoundly upregulated than miR-155 or miR-214 in NSCLC (Figure 2A–2C). Accordingly, the expression of *PTEN* was repressed more significantly than the expression of *SOCS1* or *SOCS6* (Figure 2D–2F).

### Correlation between the expression of miR-21, miR-155, miR-214, and their target genes, *SOCS1*, *SOCS6*, and *PTEN*, in 80 clinical NSCLC samples

**Figure 3:**
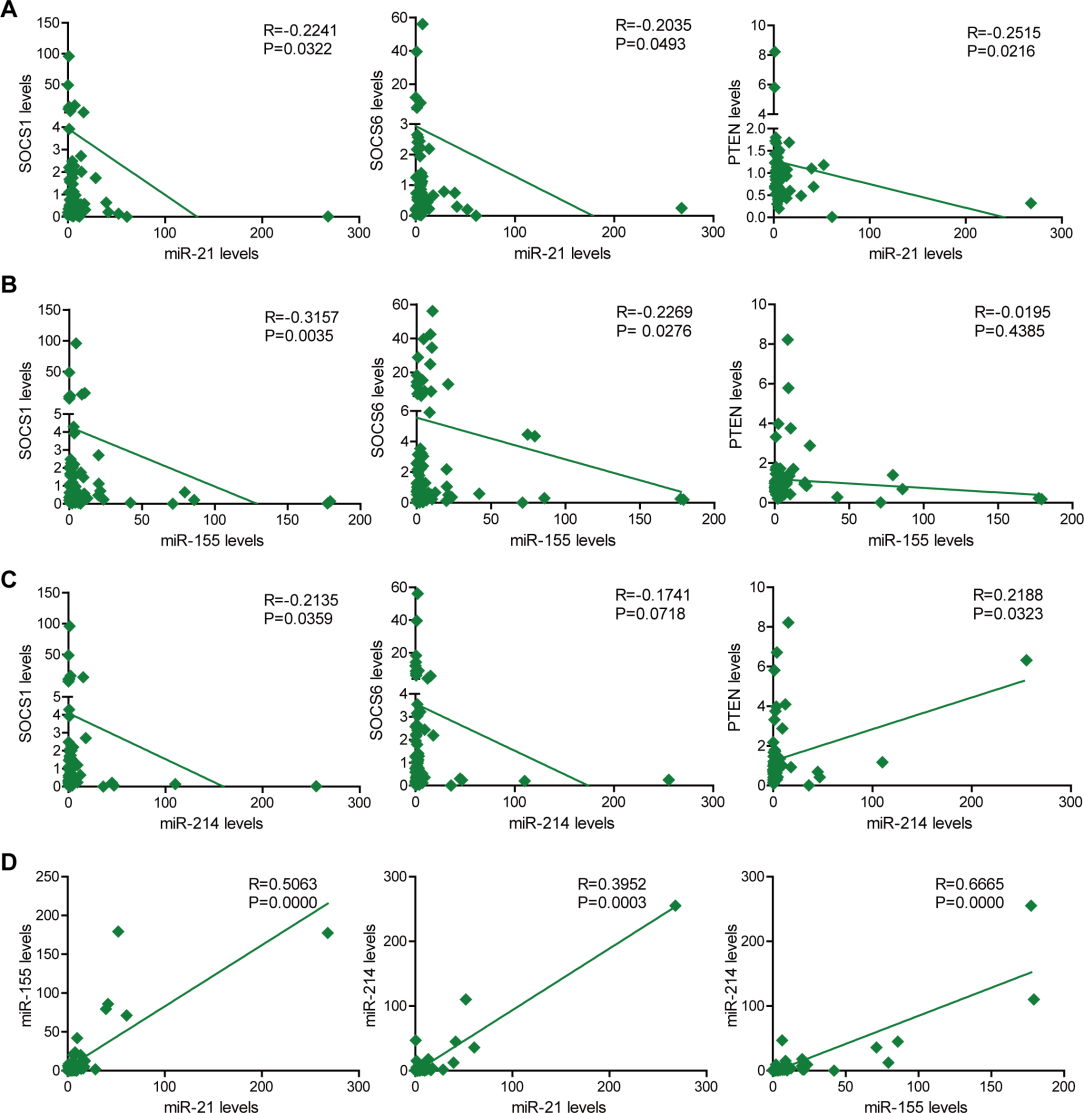
The correlation of miR-21, miR-155 and miR-214 expression with *SOCS1*, *SOCS6*, and *PTEN* expression in 80 NSCLC clinical samples (**A**) The correlation of miR-21 expression with *SOCS1*, *SOCS6*, and *PTEN* expression in 80 clinical NSCLC samples. (**B**) The correlation of miR-155 expression with *SOCS1*, *SOCS6* and *PTEN* expression in 80 clinical NSCLC samples. (**C**) The correlation of miR-214 expression with *SOCS1*, *SOCS6* and *PTEN* expression in 80 clinical NSCLC samples. (**D**) The correlation of miR-21 and miR-155 expression; miR-21 and miR-214 expression; and miR-155 and miR-214 expression in 80 clinical NSCLC samples.

As shown in Figure 3, miR-21 expression inversely correlated with the gene expression of *SOCS1*, *SOCS6*, and *PTEN* (Figure 3A). Similarly, miR-155 expression negatively correlated with the levels of *SOCS1*, *SOCS6*, and *PTEN* (Figure 3B). While miR-214 levels negatively correlated with *SOCS1* and *SOCS6* levels, we observed a positive correlation between miR-214 levels and *PTEN* expression (Figure 3C). This result may imply that miR-214 does not regulate *PTEN* expression. Positive correlations in the expression of miR-21, miR-155, and miR-214 were also detected (Figure 3D).

### Direct downregulation of *SOCS1, SOCS6*, and *PTEN* expression by miR-21 and miR-155

**Figure 4:**
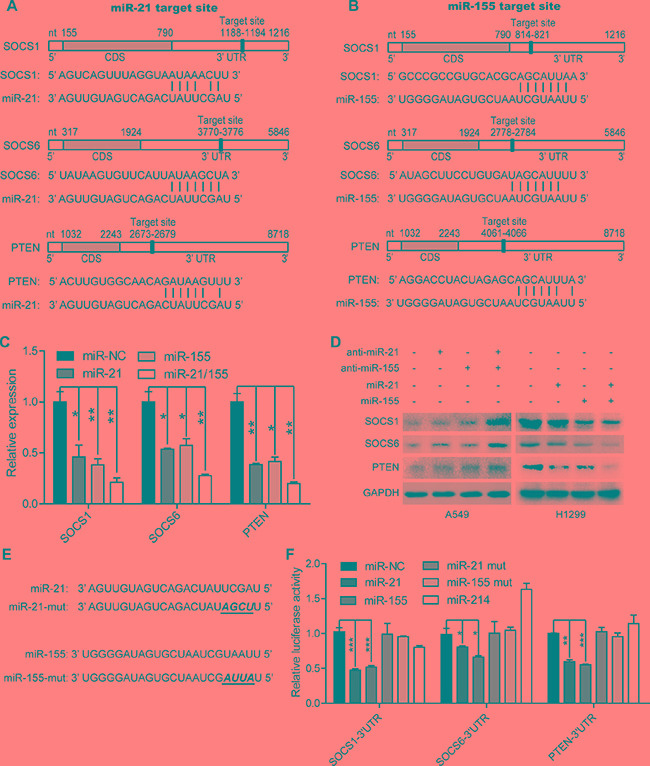
MiR-21 and miR-155 downregulate the expression of *SOCS1*, *SOCS6*, and *PTEN* in a direct manner (**A**–**B**) The predicted binding sites of miR-21 (A) and miR-155 (B) in the 3′-UTRs of *SOCS1*, *SOCS6*, and *PTEN*. (**C**)The mRNA levels of *SOCS1*, *SOCS6*, and *PTEN* in H1299 cells treated with 100 nM miR-NC, miR-21, miR-155, or combined miR-21 and miR-155 mimics.*p < 0.05, **p < 0.01. (**D**) The protein levels of SOCS1, SOCS6, and PTEN in A549 and H1299 cells treated with 100 nM miR-21, or miR-155, or miR-21/155 antisense or mimic sequences for 48 hours. (**E**) The mutant sequences of the miR-21 and miR-155 mimics. (**F**) The luciferase activity of the *SOCS1*, *SOCS6*, and *PTEN* 3′-UTR reporters in H1299 cells transfected with 100 nM miR-NC, miR-21, miR-155, miR-21/155, or miR-214 mimics, or their mutant mimics. Cells were transfected for 48 hours with 100 ng of the psiCHECK-SOCS1 3′-UTR, or psiCHECK-SOCS6 3′-UTR, or psiCHECK-PTEN 3′-UTR reporter plasmid before they were harvested for luciferase assays.*p < 0.05, **p < 0.01, ***p < 0.001.

We next performed a luciferase 3′-UTR reporter assay, which revealed that miR-214 did not bind to *SOCS1*, *SOCS6*, or *PTEN* (Figure 4F). Therefore, we excluded miR-214 from our subsequent studies, and instead investigated miR-21, miR-155 and their target genes. An analysis using TargetScan and the Miranda database revealed that the 3′-UTRs of *SOCS1*, *SOCS6* and *PTEN* contained putative binding sites for both miR-21 and miR-155 (Figure 4A–4B). Thus, we overexpressed miR-21, miR-155 or both, and found that overexpression of these miRNAs markedly inhibited the mRNA levels of *SOCS1*, *SOCS6* and *PTEN* (Figure 4C). Furthermore, inhibitors of miR-21 or miR-155 significantly increased the protein levels of SOCS1, SOCS6 and PTEN, while miR-21 or miR-155 mimics reduced the expression of these proteins (Figure 4D). More importantly, the combination of miR-21 and miR-155 mimics or inhibitors profoundly repressed or elevated (respectively) the expression of SOCS1, SOCS6 and PTEN, whereas single modification of miR-21 or miR-155 moderately affected the expression of SOCS1, SOCS6, and PTEN.

Treatment with miR-21 or miR-155 mimics reduced the luciferase activities of *SOCS1*, *SOCS6* and *PTEN* reporter constructs containing the predicted binding sites of miR-21 or miR-155. On the other hand, mimics containing four point mutations in the seed sequences of miR-21 or miR-155 did not change the luciferase activities of the *SOCS1*, *SOCS6*, and *PTEN* 3′-UTR reporters. These results demonstrate that miR-21 and miR-155 downregulate the expression of *SOCS1*, *SOCS6*, and *PTEN* by binding to their 3′-UTRs.

### Combined miR-21 and miR-155 inhibitors inhibit lung cancer cell growth more significantly than single miR-21 or miR-155 inhibitors *in vitro* and *in vivo*

**Figure 5:**
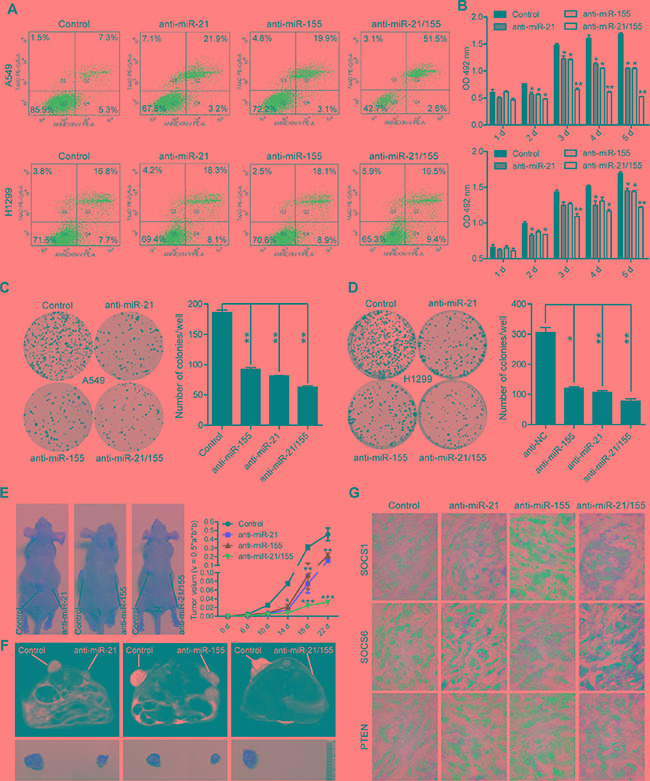
Anti-miR-21, anti-miR-155, or combined anti-miR-21 and anti-miR-155 treatment inhibits lung cancer cell growth *in vitro* and *in vivo* (**A**) The apoptosis rates of A549 and H1299 cells treated with 100 nM miR-NC, miR-21, miR-155, or miR-21/155 antisense for 24 hours. (**B**) The proliferation rates of A549 and H1299 cells treated with 100 nM miR-NC, miR-21, miR-155, or miR-21/155 antisense at different time points, as analyzed by the 3-(4,5-Dimethylthiazol-2-yl)-2,5-Diphenyltetrazolium Bromide (MTT) assay.*p < 0.05, **p < 0.01. (**C**–**D**) Colony formation assay of A549 (C) and H1299 (D) cells treated with 100 nM miR-NC, miR-21, miR-155, or miR-21/155 antisense. Colony numbers were counted after 10 days.*p < 0.05, **p < 0.01. (**E**) Tumor growth rate in mice injected with A549 cells transfected with miR-NC, miR-21, miR-155, or miR-21/155 antagomirs. (**F**) The tumor image of (E) analyzed with a nuclear magnetic resonance (MRI) assay. (**G**) Immunohistochemical analysis of the protein levels of SOCS1, SOCS6, and PTEN in tumor samples derived from mice implanted with A549 cells pre-treated with miR-NC, miR-21, miR-155, or miR-21/155 antagomirs.

Given that SOCS1, SOCS6, and PTEN are known to be critical tumor suppressors, we reasoned that restoring their expression by downregulating miRNA expression could be a good cancer treatment strategy. Treatment with miR-21 and miR-155 antisense sequences induced the apoptosis of A549 and H1299 cells (Figure 5A). In addition, the cell proliferation rate of A549 and H1299 cells was significantly inhibited, as shown by their reduced cell numbers, viability, and colony formation (Figure 5B–5D). Furthermore, tumor growth was suppressed in mice injected with A549 cells transfected with miR-21, miR-155, or miR-21/155 antagomirs (Figure 5E–5F). Accordingly, elevated protein levels of SOCS1, SOCS6, and PTEN were detected in xenografted tumors from cells treated with miR-21, miR-155, or miR-21/155 antagomirs (Figure 5G). Notably, combination treatment with miR-21 and miR-155 inhibitors was more effective than treatment with a single miRNA inhibitor (Figure 5E–5G, Supplementary Figure S4). Correspondingly, we found that many cancer-related pathways, including those involved in NSCLC, are connected to the common targets of miR-21 and miR-155 (Supplementary Figure S5). These data indicate that miR-21 and miR-155 are major promoters of tumor cell growth *in vitro* and *in vivo*.

### Correlation of miR-21/miR-155 and SOCS1/SOCS6/PTEN expression with overall survival and disease-free survival in NSCLC

**Figure 6:**
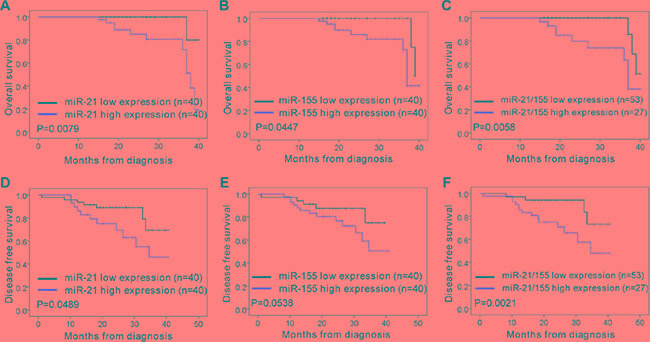
Correlation analysis of patients' overall survival and disease-free survival vs. expression of miR-21, miR-155, and miR-21/155 in NSCLC (**A**–**C**) The overall survival rates of patients with different levels of miR-21 (A), miR-155 (B), and miR-21/155 (C) in NSCLC. (**D**–**F**) The disease-free survival rates of patients with different levels of miR-21 (D), miR-155 (E), and miR-21/155 (F) in NSCLC.

**Figure 7:**
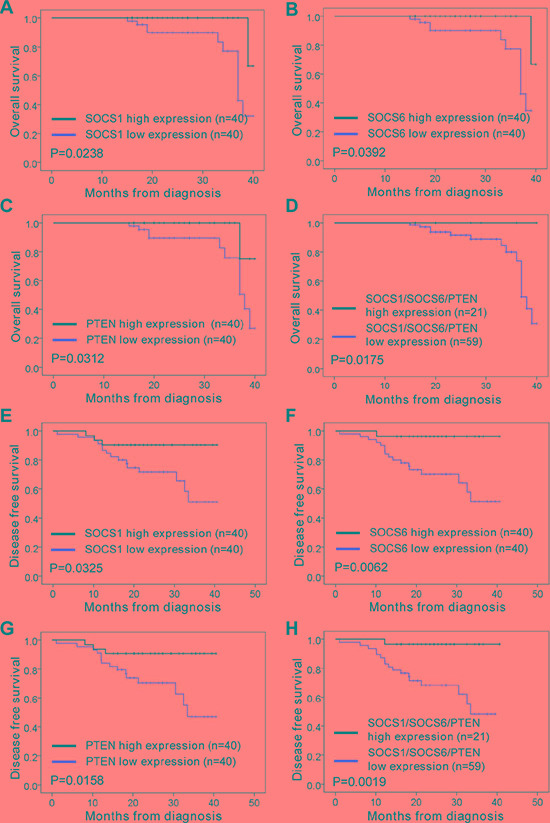
Correlation analysis of patients' overall survival and disease-free survival vs. expression of SOCS1, SOCS6, PTEN, and SOCS1/SOCS6/PTEN in NSCLC (**A**–**D**) The overall survival rates of patients with different levels of SOCS1 (A), SOCS6 (B), PTEN (C), and SOCS1/SOCS6/PTEN (D) in NSCLC. (**E**–**H**) The disease-free survival rate of patients with different levels of SOCS1 (E), SOCS6 (F), PTEN (G), and SOCS1/SOCS6/PTEN (H) in NSCLC.

In order to analyze the association of miR-21 and miR-155 levels with NSCLC disease progression in cancer patients, we analyzed the miR-21 and miR-155 expression profiles of normal human controls or cancer patients with adenocarcinoma and squamous cell carcinoma, using the parameters of overall survival (OS) and disease-free survival (DFS). When patient samples were stratified by a median cutoff of miR-21 or miR-155 expression, patients with higher miR-21 or miR-155 expression (> median) were found to have shorter OS and DFS than those expressing lower levels of miR-21 or miR-155 (≤ median) (Figure 6A–6B, 6D–6E), indicating that the levels of miR-21 and miR-155 inversely correlate with disease progression. The survival rate was significantly lower among patients overexpressing both miR-21 and miR-155 than among patients overexpressing either miR-21 or miR-155 alone (Figure 6C, 6F). By the same token, patients with lower SOCS1, SOCS6, and PTEN expression had shorter OS and DFS than those expressing higher levels of SOCS1, SOCS6, and PTEN (Figure 7A–7C, 7E–7G). Likewise, patients with combined lower expression of SOCS1, SOCS6, and PTEN had lower survival rates than those with lower expression of SOCS1, SOCS6 or PTEN alone (Figure 7D, 7H).

## DISCUSSION

MiR-21 and miR-155 are the most highly expressed miRNAs in NSCLC [10, 11, 17–20]. Thus, understanding the molecular regulation of miR-21 and miR-155 is of great value for the future development of novel therapeutic strategies. We found that miR-21 and miR-155 share nearly 30% of their predicted targets. In addition, we demonstrated that both miR-21 and miR-155 can directly inhibit the expression of *SOCS1*, *SOCS6* and *PTEN* by binding their 3′-UTRs, uncovering a new layer of the molecular mechanism whereby these genes are repressed in NSCLC.

SOCS1, SOCS6 and PTEN have been identified as tumor suppressors in a broad range of human malignancies, including NSCLC [5–8, 16, 21–27]. A previous study indicated that *SOCS1* is an evolutionarily conserved target of miR-155 in breast cancer cells [28]. Zhang et al. found that miR-21 promotes the development of NSCLC by suppressing the PTEN signaling pathway [29]. In addition, Li et al. discovered a novel miR-21-*SOCS6* axis, which might be an important way that miR-21 promotes the growth and invasion of HCC cells [30]. However, it was not certain whether miR-21 or miR-155, alone or in combination, inhibit gene expression relevant to NSCLC progression. It was even less clear whether they achieve such effects by inhibiting *SOCS1*, *SOCS6*, or *PTEN*. Here, we demonstrated that the gene expression of *SOCS1*, *SOCS6*, and *PTEN* can be downregulated by miR-21 or miR-155 alone, in a direct manner. We also demonstrated that combined treatment with miR-21 and miR-155 inhibitors or mimics more strongly affected target gene expression than single miR-21 or miR-155 inhibitors or mimics, in both A549 cells and H1299 cells (Figure 4, Figure 5). Furthermore, the survival rate was significantly lower among patients with high expression of both miR-21 and miR-155 than among patients with high expression of either miR-21 or miR-155 alone (Figure 6C, 6F).

As mentioned above, our comprehensive analysis of target genes revealed that miR-21 and miR-155 share nearly 30% of their targets. Pathway analysis demonstrated that most of the common target genes were related to cancer, including NSCLC. A previous report indicated that miR-214 regulates the acquired resistance to gefitinib in HCC827 via the PTEN/AKT signaling pathway [13]. However, our results demonstrated that miR-214 does not regulate the expression of *PTEN*, *SOCS1*, or *SOCS6* (Figures 3C and 4F). This discrepancy may be due to differences in experimental approaches or cell systems.

In conclusion, our findings have revealed that miR-21 and miR-155 promote the development of NSCLC by downregulating *SOCS1*, *SOCS6*, and *PTEN*. How miR-21 and miR-155 regulate the expression of other target genes in the progression of NSCLC or in a broad scope of cancers remains to be investigated in the near future.

## MATERIALS AND METHODS

### Human NSCLC tissues

Eighty paired normal and NSCLC tissues were obtained from Shanghai 10th People's Hospital for diagnostic purposes. The local ethics committee approved the study, and the regulations of this committee were followed. Written consent was obtained from patients.

### Mice

Male BALB/c nude mice were purchased from Beijing Vital River Laboratory Animal Inc., Beijing. All animals were housed and maintained under pathogen-free conditions. All animal experiments were performed in compliance with the guide for the care and use of laboratory animals and were approved by the institutional biomedical research ethics committee of the Beijing Institutes for Biological Sciences, Chinese Academy of Sciences.

### Cell lines, plasmids and transfection

Human NSCLC cell lines, A549 and H1299, were purchased from ATCC. Cells were cultured in RPMI1640 or DMEM containing 10% fetal bovine serum (HyClone) and 1% penicillin/streptomycin (Invitrogen).

Transfection of cell lines was performed with Lipofectamine 2000 reagent (Invitrogen), according to the manufacturer's instructions. To inhibit or overexpress miR-21 and miR-155, we transfected 100 nM antisense or mimic sequences (respectively) of miR-21, miR-155 or both (miR-21/155) into A549 or H1299 cell lines for 48 hours. Human *SOCS1*, *SOCS6* and *PTEN*3′-UTR sequences were cloned into a psiCHECK^TM^-2 vector.

### Western blot

Proteins were extracted from cells with 1× loading lysis buffer, and their concentrations were measured with a Lowry protein assay. Western blot analysis was performed according to a previously described standard method [31] with anti-SOCS1 (1:500, sc-9021, Santa Cruz Biotechnology), anti-SOCS6 (1:500, sc-5608, Santa Cruz Biotechnology), anti-PTEN (1:500, #9559, Cell Signaling Technology) or anti-GAPDH (1:2000, Sigma) antibodies.

### RNA extraction and real-time qRT-PCR

Total miRNAs were isolated from cultured cells or surgically resected from fresh NSCLC tissues with a mirVana miRNA Isolation Kit (Ambion) in accordance with the manufacturer's instructions. Total RNAs were isolated from cell lines with TRIzol Reagent (Invitrogen, 15596-018) as described in the manufacturer's protocols. To obtain cDNA, we performed a reverse transcription reaction with Transcript First Strand Synthesis Supermix (TransGen Biotech, AT301) according to the manufacturer's instructions, using 1 μg total RNAs as the template. For miRNA, 0.5 μg total RNA from each sample was reverse-transcribed to cDNA by means of specific miRNA stem loop primers. All quantitative real-time reverse transcription PCR (qRT-PCR) was performed on a 7500 Fast Real-Time PCR System (Applied Biosystems), and all qRT-PCR reagents and consumables were purchased from Applied Biosystems and TaKaRa. The mRNA and miRNA levels were quantitatively assessed by SYBR Green-based qRT-PCR with gene-specific primers. The sequences of primers are listed in Supplementary Table S1. *GAPDH* and *U6* were used as internal normalization controls.

### Luciferase reporter transfection and dual luciferase assay

In the 3′-UTR reporter assay, H1299 cells were grown to 80%–90% density in 24-well plates and transfected with 100ng of the psiCHECK^TM^-2-SOCS1-3′-UTR, psiCHECK^TM^-2-SOCS6-3′-UTR, or psiCHECK^TM^-2-PTEN-3′-UTR, along with 100 nM of various mimics (Genepharma) and 1 μl of Lipofectamine 2000. Lysates were harvested 48 hours after transfection, and reporter activities were measured with a Dual Luciferase Assay (Promega) according to protocols supplied by the manufacturer.

### Immunohistochemistry

Formalin-fixed, paraffin-embedded tumor tissue blocks from mice were used in our investigation. Immunohistochemical staining was performed with various primary antibodies and HRP-conjugated secondary antibodies. The antibodies included anti-SOCS1 (1:100, sc-9021, Santa Cruz Biotechnology), anti-SOCS6 (1:100, sc-5608, Santa Cruz Biotechnology) and anti-PTEN (1:100, #9559, Cell Signaling Technology).

### Flow cytometry

Cells were seeded in six-well plates at a density of 1 × 10^5^ cells/well. After 48 hours, the cells were detached with 0.25% trypsin and washed. PE-AnnexinV (BD Biosciences) and 7-AAD (BD Biosciences) were added for 30 minutes at 4°C in the dark prior to flow cytometry analysis. The data were analyzed with FlowJo 7.6 software (TreeStar, Inc.).

### WST-1 assay

A549 cells and H1299 cells were seeded at a density of 1 × 10^3^ cells/well in 96-well plates and cultured at 37°C in a 5% CO_2_ incubator. The cell proliferation rate was measured with a WST-1 Cell Proliferation and Cytotoxicity Assay Kit (Beyotime, C0036) 1, 2, 3, 4, and 5 days after seeding, according to the manufacturer's protocol.

### Colony formation

A total of 1 × 10^3^ cells were seeded in 60-mm cell culture dishes. After 10 days, cells were stained with Crystal Violet Staining Solution (Beyotime, C0121), and colonies containing ≥ 20 cells were counted. Representative colonies were photographed with a Canon EOS 50.

### Tumorigenesis assay

All experimental procedures were approved by the Institutional Animal Care and Use Committee of the Central Laboratory of Beijing Shijitan Hospital, Capital Medical University. BALB/c nude mice (6–7 weeks of age, 18–20 g, male) were randomly divided into three groups (*n* = 6 per group). Negative control miRNA (miR-NC), miR-21, miR-155, or miR-21/155 antagomirs (all at 100 nM concentration) were first transfected into A549 cells for 48 hours. Then, 1 × 10^6^ transfected cells in 0.1 mL of medium were subcutaneously injected into the mice at left or right back area. The mice were sacrificed 3–4 weeks after injection, and tumors were removed, fixed, and processed for sectioning.

## SUPPLEMENTARY MATERIALS FIGURES AND TABLES


