# Supplementary material for: MiR-21 and MiR-155 promote non-small cell lung cancer progression by downregulating SOCS1, SOCS6, and PTEN
Source: Oncotarget. 2016 Nov 2;7(51):84508–19. doi: 10.18632/oncotarget.13022 (PMC5356677; doi:10.18632/oncotarget.13022)
Supplement: Supplementary file 1 [file oncotarget-07-84508-s001.pdf]

MiR-21 and MiR-155 promote non-small cell lung cancer progression by downregulating *SOCS1*, *SOCS6*, and *PTEN*

Supplementary Materials

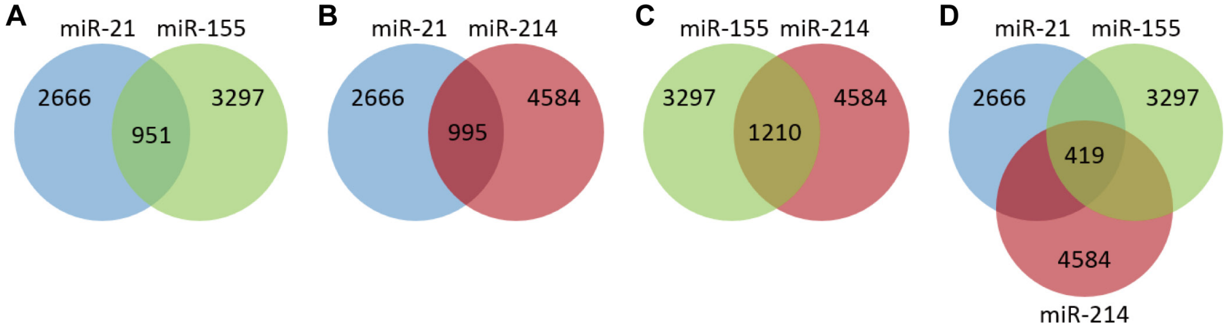

Supplementary Figure S1: The overlapping predicted targets of miR-21, miR-155, and miR-214. (**A**) The overlapping predicted targets of miR-21 and miR-155. (**B**) The overlapping predicted targets of miR-21 and miR-214. (**C**) The overlapping predicted targets of miR-155 and miR-214. (**D**) The overlapping predicted targets of miR-21, miR-155 and miR-214.

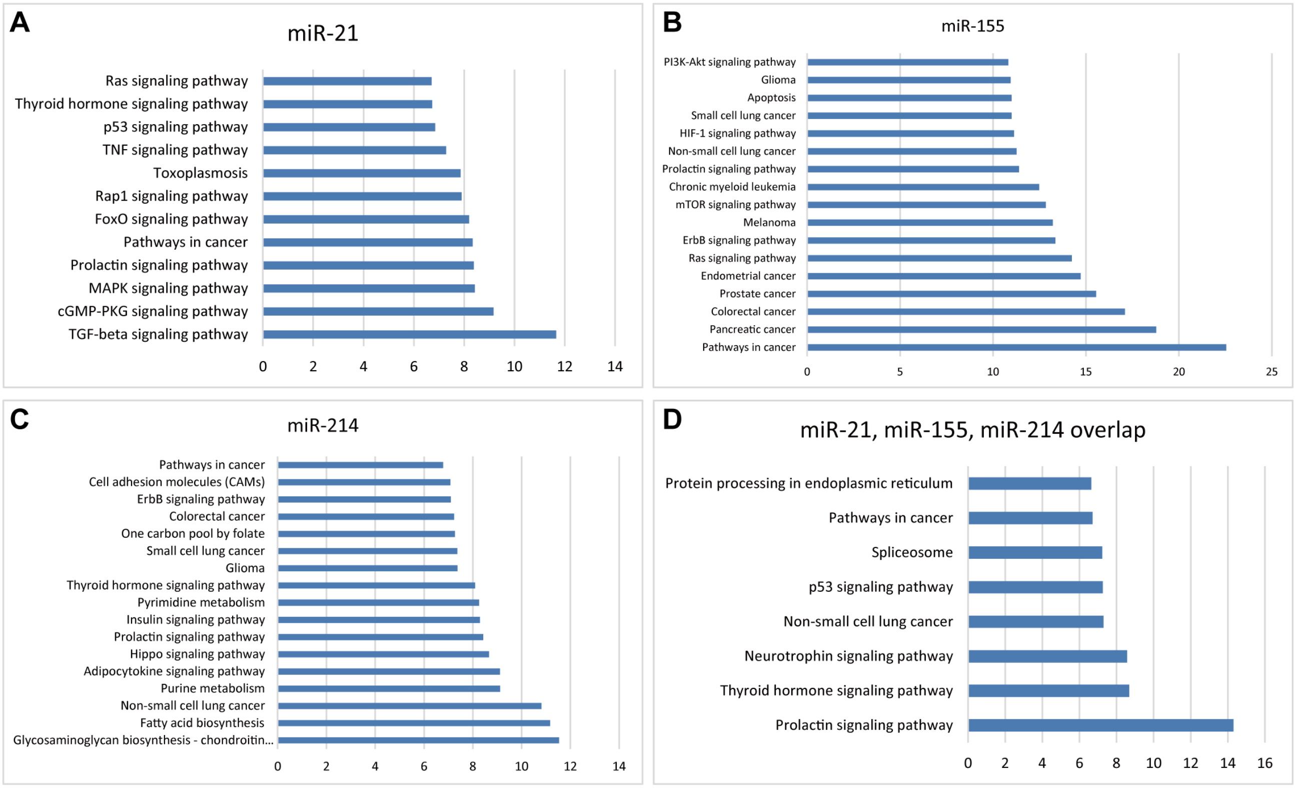

Supplementary Figure S2: Pathway analysis of the predicted targets of miR-21, miR-155 and miR-214, and of the overlapping predicted targets of miR-21/155/214. (A–C) Pathway analysis of the predicted targets of miR-21 (A), miR-155 (B) and miR-214 (C). (**D**) Pathway analysis of the overlapping predicted targets of miR-21/155/214.

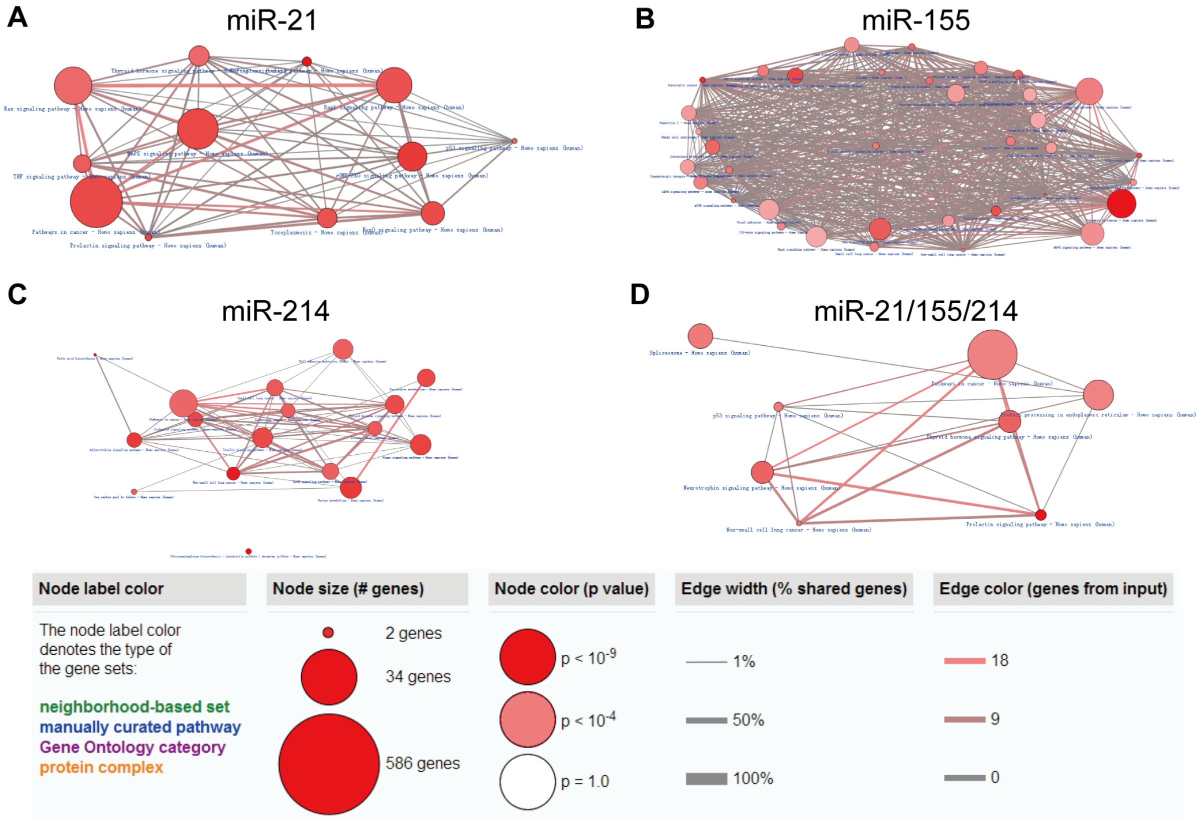

Supplementary Figure S3: The regulation networks of the predicted targets of miR-21, miR-155, miR-214, and of the overlapping predicted targets of miR-21/155/214. (**A**) The regulation network of the predicted targets of miR-21. (**B**) The regulation network of the predicted targets of miR-155. (**C**) The regulation network of the predicted targets of miR-214. (**D**) The regulation network of the overlapping predicted targets of miR-21/155/214.

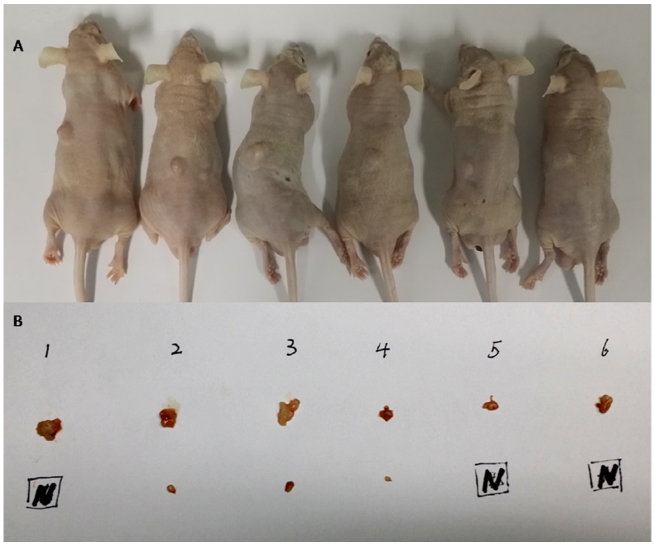

Supplementary Figure S4: Treatment with miR-21 and miR-155 inhibitors obviously inhibited tumor growth. (**A**) Treatment with miR-21 and miR-155 inhibitors obviously inhibited tumor growth *in vivo*. (**B**) Tumor size was measured after dissection.

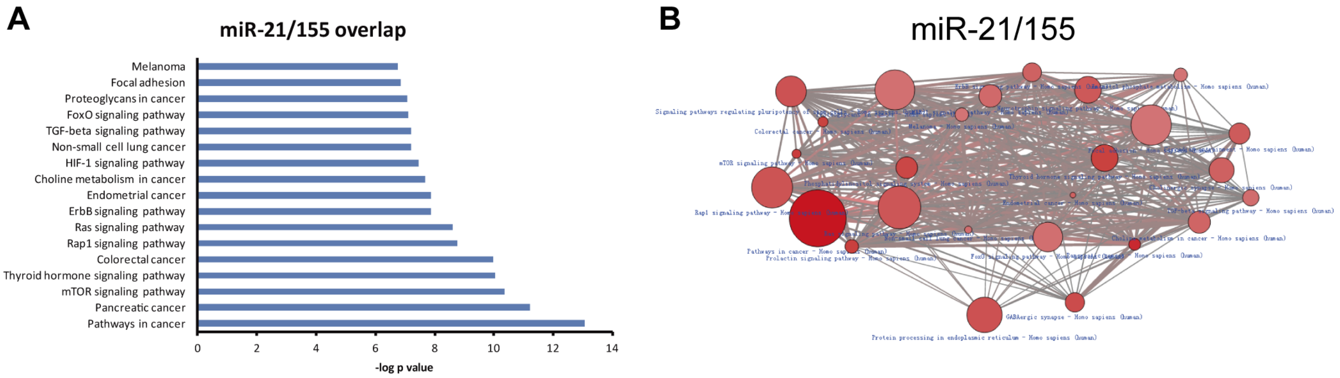

Supplementary Figure S5: Pathway analysis and regulation network of the overlapping predicted targets of miR-21 and miR-155. (**A**) Pathway analysis of the overlapping predicted targets of miR-21/155. (**B**) The regulation network of the overlapping predicted targets of miR-21/155.

Supplementary Table S1: The sequences of primers of miRNAs and genes real time PCR

Name		5′–3′
U6 RT		GTCGTATCCAGTGCAGGGTCCGAGGTATTCGCACTGGATACGA CAAAAAT
hsa-miR-21 RT		GTCGTATCCAGTGCAGGGTCCGAGGTATTCGCACTGGATACGAC TCAACA
hsa-miR-155 RT		GTCGTATCCAGTGCAGGGTCCGAGGTATTCGCACTGGATACGAC ACCCCT
hsa-miR-214 RT		GTCGTATCCAGTGCAGGGTCCGAGGTATTCGCACTGGATACGAC ACTGCC
U6 real time PCR	Forward	CGGCGGTCGTGAAGCGTTCCAT
	Reverse	CCAGTGCAGGGTCCGAGGTAT
hsa-miR-21 real time PCR	Forward	CGGCGGTTAGCTTATCAGACTGA
	Reverse	CCAGTGCAGGGTCCGAGGTAT
hsa-miR-155 real time PCR	Forward	CGGCGGTTTAATGCTAATCGTGAT
	Reverse	CCAGTGCAGGGTCCGAGGTAT
hsa-miR-214 real time PCR	Forward	CGGCGGTACAGCAGGCACAGACA
	Reverse	CCAGTGCAGGGTCCGAGGTAT
SOCS1 real time PCR	Forward	GTAGGAGGTGCGAGTTCAGG
	Reverse	GACCCCTTCTCACCTCTTGA
SOCS6 real time PCR	Forward	AGGAGCAGAGTTCGGATCAA
	Reverse	ACCATTGCTACCTCCAATGC
PTEN real time PCR	Forward	TTGGCGGTGTCATAATGTCT
	Reverse	GCAGAAAGACTTGAAGGCGTA
